# Interleukin-13 Alters Tight Junction Proteins Expression Thereby Compromising Barrier Function and Dampens Rhinovirus Induced Immune Responses in Nasal Epithelium

**DOI:** 10.3389/fcell.2020.572749

**Published:** 2020-09-25

**Authors:** Zhi-Qun Huang, Jing Liu, Hsiao Hui Ong, Tian Yuan, Xiang-Min Zhou, Jun Wang, Kai Sen Tan, Vincent T. Chow, Qin-Tai Yang, Li Shi, Jing Ye, De-Yun Wang

**Affiliations:** ^1^Department of Otolaryngology-Head and Neck Surgery, First Affiliated Hospital of Nanchang University, Jiangxi, China; ^2^Department of Otolaryngology, Yong Loo Lin School of Medicine, National University of Singapore, National University Health System, Singapore, Singapore; ^3^Department of Otolaryngology-Head and Neck Surgery, Affiliated Hospital of Jiujiang University, Jiangxi, China; ^4^Department of Otorhinolaryngology-Head and Neck Surgery, Third Affiliated Hospital, Sun Yat-sen University, Guangzhou, China; ^5^Department of Otolaryngology, Second Hospital of Shandong University, Jinan, China; ^6^NUHS Infectious Diseases Translational Research Program, Department of Microbiology and Immunology, Yong Loo Lin School of Medicine, National University of Singapore, Singapore, Singapore

**Keywords:** chronic rhinosinusitis with nasal polyps, human nasal epithelial cells, interleukin-13, rhinovirus, tight junctions

## Abstract

Tight junctions (TJs) are intercellular structures which are essential for epithelial barrier function and play an important role in antimicrobial defense. Epithelium dysfunction and type-2-skewed inflammation are two main pathological phenomena of chronic rhinosinusitis with nasal polyps (CRSwNP). However, the effect of pro-inflammatory type-2 cytokine IL-13 on TJs in CRSwNP is poorly understood. Nasal biopsies of CRSwNP patients and *in vitro* IL-13-matured human nasal epithelial cells (hNECs) were used to analyze epithelial markers and TJ proteins. Epithelium permeability, transepithelial electrical resistance (TEER), expression of TJs were quantified for IL-13-matured hNECs and that with RV infection. The expression of occludin, claudin-3, and ZO-1 were significantly decreased in CRSwNP biopsies and in hNECs after IL-13 treatment. IL-13 treatment increased epithelium permeability, decreased TEER and altered hNECs composition resulting in lesser ciliated cells and mucus over-secretion. Interestingly, claudin-3 is selectively expressed on ciliated cells. While RV infection induced minimal changes to TJs, the IL-13-matured hNECs has reduced capacity for upregulation of *IFN-*λ*1* and *CXCL10* but further increased the expression of *TSLP* upon RV infection. These findings suggested that IL-13-mediated dysfunction of TJs and compromised epithelial barrier. IL-13-induced cilia loss conferred lowered viral replication and impaired antiviral responses of nasal epithelium against RV infection.

## Introduction

The normal sinonasal epithelium comprises of ciliated cell, goblet cell, club cell, and basal cell (Heffler et al., 2018). Tight junctions (TJs), which are located at the most apical part of intercellular junction between these epithelial cells, serve as a physical barrier of nasal airway epithelium to protect it from the external environment (Holgate, 2007). The main components of TJs are occludin, claudins, junctional adhesion molecules and scaffold protein zonula occludens (ZO). These TJ proteins are the key structural proteins which maintain epithelial polarity, regulate pericellular permeability and participate in epithelial cell proliferation, differentiation and migration (Kojima et al., 2013; Zihni et al., 2014). These functions play an important role in distinct tissue compartmentalization and homeostasis of nasal epithelium. Disruption of TJs may cause reduction in epithelial cohesion and integrity which may lead to a variety of pathological conditions. It has been demonstrated that TJ proteins are involved in the pathophysiology of chronic airway inflammatory disorders including chronic rhinosinusitis with nasal polyps (CRSwNP), allergic rhinitis and asthma (Steelant et al., 2016; Looi et al., 2018; Tian et al., 2018).

IL-13 is the key regulator in type-2 mediated inflammation in CRSwNP. IL-13 induces goblet cell hyperplasia, loss of cilia, inducible nitric oxide syntheses production and fibrosis to accelerate inflammation and promote remodeling (Fulkerson et al., 2006; Doran et al., 2017). The elevated levels of pro-inflammatory cytokines contributing to diseases pathophysiology and barrier dysfunctions have been implicated in a variety of tissues (Fulkerson et al., 2006; Saatian et al., 2013; Bauer et al., 2014). Studies have shown that IL-13 impaired barrier function by reducing expression of occludin, ZO-1 and β-catenin in primary bronchial epithelial cells of asthmatic patients (de Boer et al., 2008) and also disrupted intestinal barrier by upregulation of claudin-2 (Luettig et al., 2015). Additionally, it was found that with IL-4/IL-13 and IL-5 stimulation, E-cadherin and ZO-1 were downregulated in cultured epithelial cells of patients with allergic rhinitis (Lee et al., 2016). Hence, these studies further support the hypothesis that change in expression and localization of TJs accounts for epithelial barrier dysfunctions in chronic inflammatory diseases.

In CRSwNP, persistent and prolonged mucosal inflammation is well characterized and is closely related to elevated type-2 cytokines. However, the role of these cytokines on impairing the nasal epithelial barrier is still unknown. On the other hand, human nasal epithelial cells (hNECs), being the primary entry point of most inhaled pathogens, are the key players in regulating inflammatory responses and are an important source of pro-inflammatory cytokines (Loxham et al., 2014). Among respiratory viruses, rhinovirus (RV) is most commonly associated with exacerbation of chronic airway disease (Sajjan et al., 2008; Kennedy et al., 2012). It was found that RV infection damaged TJs integrity in airway epithelial cells of asthmatic patients by reducing ZO-1, occludin and claudin-1 protein expression (Looi et al., 2018). In addition, while it is reported that RV caused transient barrier disruption in a model of normal air-liquid interface (ALI) differentiated airway epithelium, RV infection at initial stage of differentiation in an injury model prolonged barrier dysfunction by decreasing transepithelial electrical resistance (TEER) and occludin level (Faris et al., 2016). Furthermore, recent study has shown that RV infection increased production of pro-inflammatory mediators and contributed to the exaggerated inflammatory response in *in vitro* cancer cell line (Herbert et al., 2017). While RV is reported to be highly prevalent in chronic rhinosinusitis patients (Cho et al., 2013), the underlying mechanism of the association of airway disease with chronic inflammation and virus comorbidity is still poorly understood.

Our previously established *in vitro* IL-13-matured hNECs model using IL-13 stimulation closely mimicked the physiological condition and epithelium responses of *in vitro* nasal mucosa in CRSwNP (Liu et al., 2018). Using this model, we investigate the direct effect of IL-13 on hNECs and TJ proteins expression in pseudostratified layers to analyze the nasal epithelial barrier functions. Moreover, to better define the effects of respiratory viruses on TJs of inflammatory airway model, we have also analyzed the nasal epithelial barrier integrity, remodeling and immune responses of IL-13-treated hNECs against RV acute infection.

## Results

### Aberrant Expression and Association of TJs and Epithelial Cell Markers in Nasal Biopsy Specimens of CRSwNP Patients

**FIGURE 1:**
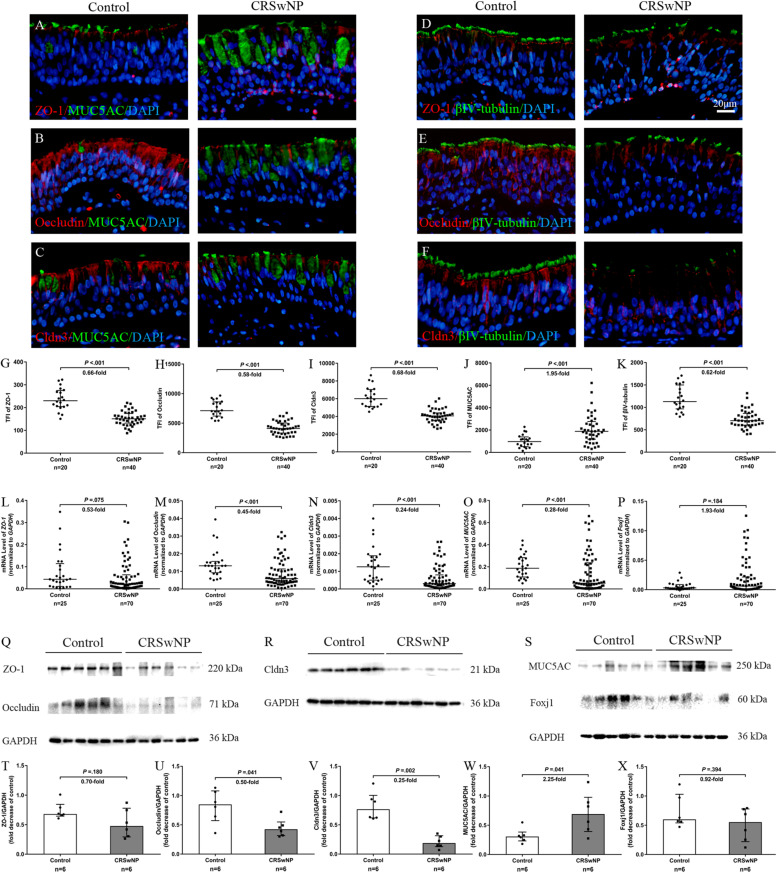
Aberrant expression of TJs and epithelial cell markers in nasal biopsy specimens of CRSwNP patients. Representative IF staining of ZO-1 **(A,D)**, occludin **(B,E)**, Cldn3 **(C,F)** with MUC5AC **(A–C)** and βIV-tubulin **(D–F)** in nasal biopsies from control subjects and CRSwNP patients. TFI level of ZO-1, occludin, Cldn3, and βIV-tubulin were decreased in CRSwNP (**G–I,K,** respectively) whereas MUC5AC TFI level was increased in CRSwNP patients **(J)**. The mRNA levels of *ZO-1*, occludin, and Cldn3 were decreased in CRSwNP as compared to control subjects **(L–N)**. There was no significant change in mRNA expression of Foxj1 between two groups **(P)**. Despite higher protein expression of MUC5AC in CRSwNP, its mRNA level was lower in CRSwNP tissue **(O)**. Similarly, protein expression levels of *ZO-1*, occludin and Cldn3 were lower, and MUC5AC was higher in CRSwNP patients by western blotting assay **(Q–W)**, whereas, *Foxj1* protein level was not change between CRSwNP and control subjects **(S,X)**. The mRNA levels were quantified by RT-qPCR assays, relative expression of the target gene was normalized to 2^– ΔCT^ with *GAPDH*. Statistical analysis was calculated using the Mann–Whitney *U* test. Data were presented as median with an interquartile range. Scale bar = 20 μm.

First, we investigated the expression and localization of TJs proteins (ZO-1, occludin and Cldn3) and epithelial cell marker MUC5AC (goblet cell) and βIV-tubulin (ciliated cell) in control subjects and CRSwNP patients. The epithelium in CRSwNP patients was damage as shown by loss of cilia and abnormal epithelium remodeling (goblet cell hyperplasia) by immunofluorescence (IF) staining (Figures 1A–F,J,K). In control subjects, ZO-1 was consistently expressed and localized at superficial side of the nasal epithelium (Figures 1A,D) and occludin was localized at both superficially and suprabasally at the cell boundaries but at a higher intensity at the superficial layer (Figures 1B,E). Cldn3 was found at the cell boundaries and at the superficial layer of nasal epithelium (Figures 1C,F). On the other hand, in CRSwNP patients, protein localization of ZO-1, occludin, and Cldn3 was reduced (all *P* < 0.001; Figures 1A–I). The protein expression levels of ZO-1, occludin, and Cldn3 were decreased in CRSwNP patients by western blotting assay (*P* = 0.180, 0.041, and 0.002; Figures 1Q,R,T–V). Similarly, the mRNA expression levels of *ZO-1*, *occludin*, and *Cldn3* were reduced in CRSwNP patients as compared to the control subject (Figures 1L–N).

**FIGURE 2:**
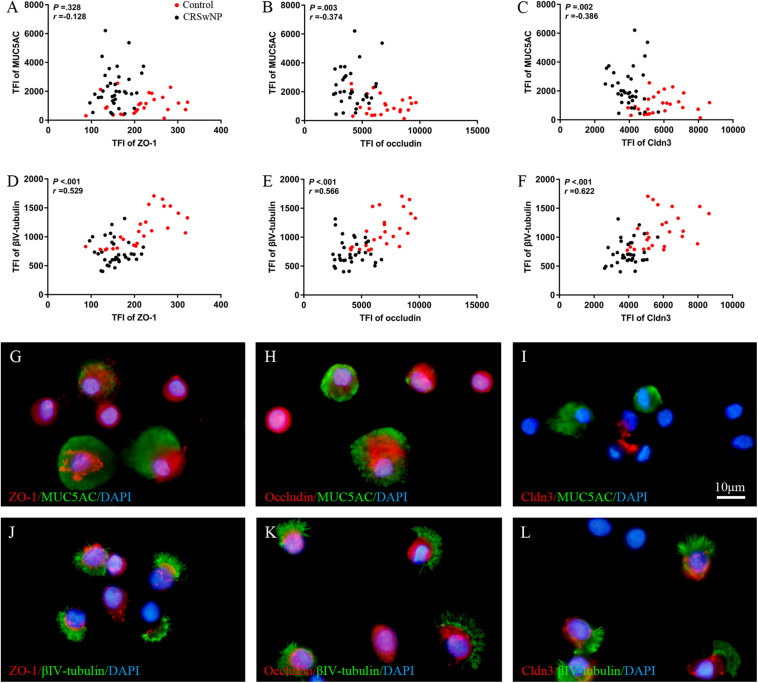
Correlation of TJs with goblet and ciliated cell in nasal biospecimen. TFI level of MUC5AC was not correlated with ZO-1 **(A)** but was negatively correlated with occludin and Cldn3 **(B,C)**. βIV-tubulin TFI level was positively correlated with ZO-1, occludin, and Cldn3 **(D–F)** in CRSwNP and control subjects. IF staining of single primary nasal cells showed that both ZO-1 **(G,J)** and occludin **(H,K)** were co-stained with goblet and ciliated cell. Interestingly, Cldn3 positive staining was only observed in ciliated cell **(L)**, but not goblet cell **(I)**. Spearman r characteristic was performed for statistical analysis. Black dot refers to CRSwNP (*n* = 40) and red dot refers to control subjects (*n* = 20). Scale bar = 10 μm.

Despite higher MUC5AC protein expression in CRSwNP, its mRNA expression was lower in tissue of CRSwNP (Figures 1J,O,S,W) while there was no significant change both in protein and mRNA expression of Forkhead box J1 (Foxj1) (Figures 1P,S,X). Furthermore, we found that MUC5AC expression was negatively correlated with occludin (*r* = −0.374, *P* = 0.003; Figure 2B) and Cldn3 (*r* = −0.386, *P* = 0.002; Figure 2C) but not with ZO-1 (*r* = −0.128, *P* = 0.328; Figure 2A) in nasal biopsies. βIV-tubulin expression was positively correlated with ZO-1 (*r* = 0.529, *P* < 0.001; Figure 2D), occludin (*r* = 0.566, *P* < 0.001; Figure 2E) and Cldn3 (*r* = 0.622, *P* < 0.001; Figure 2F). Then, we examined the expression of TJs in ciliated and goblet cells by single cell staining of primary nasal cells. We found that both ZO-1 (Figures 2G,J) and occludin (Figures 2H,K) were co-stained with goblet and ciliated cell. Interestingly, Cldn3 staining was only observed in ciliated cell (Figure 2L) but not goblet cell (Figure 2I).

Moreover, in nasal specimens, we found that the expression level of IL-13 increased significantly in CRSwNP as compared to healthy controls (Supplementary Figure E1). Taken together, a defective epithelia barrier with an altered expression pattern of TJs was observed in patients of CRSwNP and this maybe associate with alteration of nasal epithelial homeostasis during chronic airway inflammation.

### IL-13 Induces Epithelial Remodeling and Disrupts Epithelial Barrier Integrity in hNECs

**FIGURE 3:**
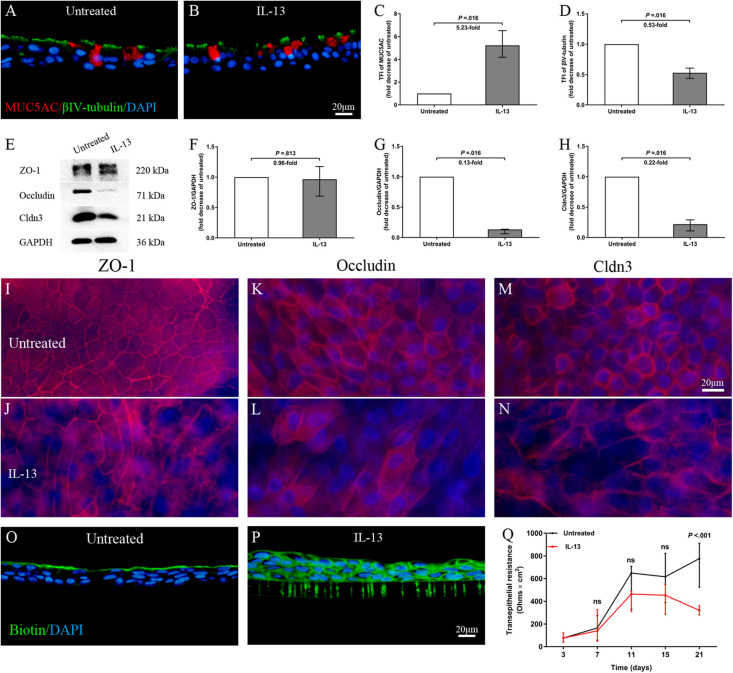
IL-13 induces epithelial remodeling and disrupts epithelial barrier integrity in hNECs. Representative IF images showed that IL-13 treatment induced more MUC5AC-secreting goblet cells and loss of ciliated cells in hNECs **(A–D)**. Western blotting results indicated that the protein expression of occludin and Cldn3 but not ZO-1 were significantly decreased with IL-13 treatment **(E–H)**. Damaged TJs proteins were shown by irregular staining patterns of ZO-1, occludin and Cldn3 in IL-13-matured hNECs as compared to untreated controls **(I–N)**. Intercellular epithelium permeability of IL-13-matured hNECs increased as compared to untreated hNECs **(O–P)**. IL-13 treatment of hNECs during differentiation also significantly reduced TEER at Day 21 of ALI culture **(Q)**. Two-way ANOVA was used to analyze differences between hNECs with and without IL-13 treatment. Data were presented as median with an interquartile range. Fold change was quantified with reference to untreated hNECs. Scale bar = 20 μm. hNECs, *n* = 7.

To analyze the direct effect of IL-13 on nasal epithelial barrier functions, IL-13 was added throughout hNECs differentiation in ALI culture. Cilia loss and mucus over-secretion were observed in IL-13-matured hNECs (all *P* = 0.016; Figures 3A–D). Similar to the trend observed in nasal biospecimen, the protein expression of occludin and Cldn3 but not ZO-1 were significantly decreased in hNECs with IL-13 treatment (all *P* = 0.016; Figures 3E–H). IL-13 also disrupted ZO-1, occludin, and Cldn3 as shown by an irregular staining patterns as compared to untreated hNECs (Figures 3I–N). In addition, epithelium permeability assay using Sulfo-NHS-Biotin as a tracer revealed that IL-13 increased the paracellular permeability of hNECs (Figures 3O,P). Long-term exposure of hNECs to IL-13 resulted in a reduction of TEER (Figure 3Q).

### IL-13 Induced Regulation of TJ Genes During hNECs Differentiation

**FIGURE 4:**
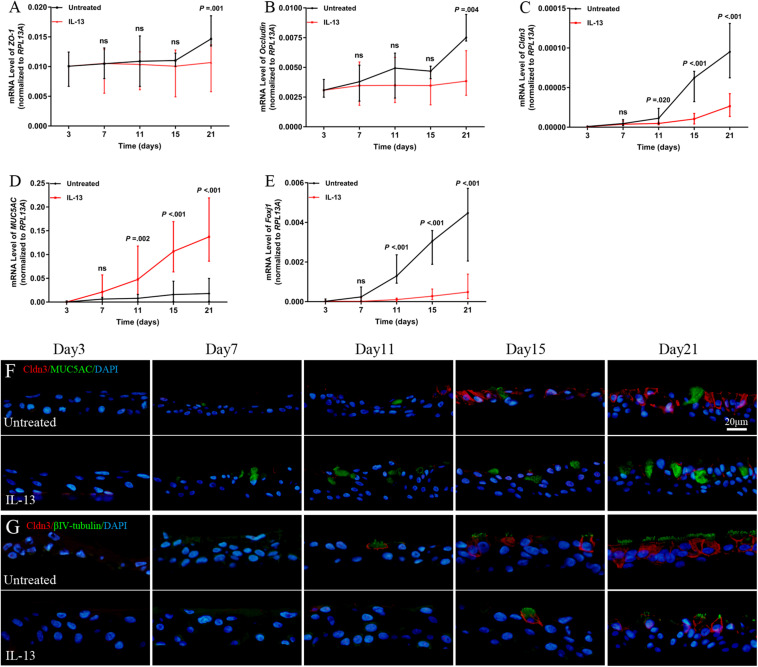
Regulation of TJs protein expression during hNECs differentiation. IL-13 decreased the mRNA expression of *ZO-1*, *occludin*, *Cldn3*, and *Foxj1* but increased *MUC5AC* mRNA level in IL-13-matured hNECs as compared to untreated group **(A–E)**. IF staining was performed for Cldn3, MUC5AC, and βIV-tubulin from Day 3 to 21 during hNECs differentiation **(F,G)**. Cldn3 was only detected when ciliated cells were first observed at Day 11 and Cldn3 was co-stained with βIV-tubulin during hNECs differentiation. Cldn3 localization is weakly detectable at poorly ciliated areas of hNECs induced by IL-13 whereas Cldn3 localization was more widespread in regions with more fully ciliated cells in untreated hNECs. The relative target gene was normalized to 2^– ΔCT^ with *RPL13A* as a housekeeping gene. Two-way ANOVA was used to analyzed differences between hNECs with and without IL-13 treatment. Data were presented as median with an interquartile range. Scale bar = 20 μm. hNECs, *n* = 9.

Next, we analyzed the role of IL-13 on regulation of the TJs formation during hNECs differentiation. *Cldn3* mRNA expression level was significantly reduced from Day 11 onward while the *ZO-1* and *occludin* mRNA expression were significantly reduced only when hNECs were fully differentiated at Day 21 (Figures 4A–C). IL-13 upregulated and downregulated mRNA expression of *MUC5AC* and *Foxj1* during hNECs differentiation, respectively (Figures 4D,E).

Positive IF staining of Cldn3 was detected when ciliated cells were first observed at Day 11 and was colocalized with βIV-tubulin-positively stained cells during differentiation of untreated hNECs, while Cldn3 staining was only detected from Day 15 onward in IL-13-matured hNECs (Figures 4F,G). In addition, there was lower expression of Cldn3 observed in IL-13-matured hNECs with less ciliated cells as compared to more widespread expression pattern of Cldn3 in untreated controls with more ciliated cells. On the other hand, positive stained ZO-1 and occludin were observed to localize at cell-to-cell contact sites as early as Day 7 and 3 at early stage of differentiation of hNECs while their localizations were non-linear and fragmented at cell-to-cell boundaries in IL-13-matured hNECs (Supplementary Figures E2A–D).

### IL-13 Modulates the Effects of RV Infection in hNECs

**FIGURE 5:**
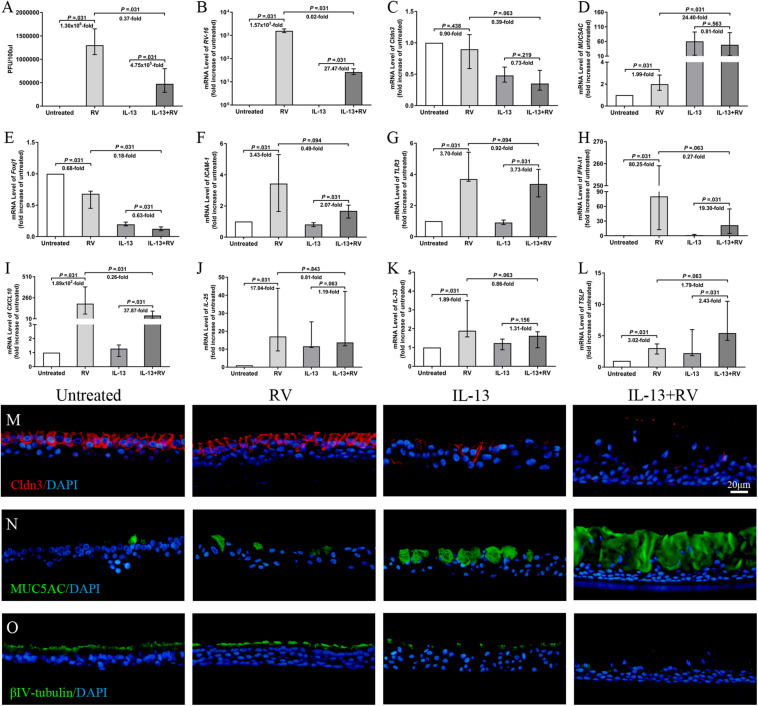
Effects of RV infection on TJs and innate immune response on IL-13-treated hNECs. The RV progeny production and viral RNA expression were significantly increased for both untreated and IL-13-treated hNECs. Extent of RV progeny production and viral RNA expression were significantly lower in IL-13-treated hNECs as compared to untreated hNECs **(A,B)**. RV infection induced trend of reduction of *Cldn3* mRNA expression in both IL-13-treated and untreated hNECs **(C)**. RV infection only significantly increased the mRNA expression of *MUC5AC* in untreated but not in IL-13-treated hNECs. RV downregulated *Foxj1* mRNA levels for both untreated and IL-13-treated hNECs. With IL-13 treatment, regulation of mRNA level of *MUC5AC* and *Foxj1* were significantly higher and lower, respectively than in untreated hNECs **(D,E)**. RV infection significantly increased the mRNA expression of RV receptor *ICAM-1*, pathogen recognition receptor *TLR3* and antiviral *IFN-*λ*1* and *CXCL10* in both untreated and IL-13-treated hNECs. With IL-13 treatment, upregulation of mRNA level of *CXCL10* was significantly lower than in untreated hNECs **(F–I)**. RV infection significantly upregulated mRNA expression of *IL-25* and *IL-33* for untreated hNECs but not IL-13-treated hNECs **(J,K)**. RV significantly regulated the mRNA expression of *TSLP* in both untreated and IL-13-treated hNECs **(L)**. IF staining showed that RV infection induced slight decrease in expression of Cldn3 in IL-13-treated hNECs while expression of MUC5AC and βIV-tubulin was, respectively higher and lower in IL-13-treated hNECs with RV infection **(M–O)**. The relative target gene was normalized to 2^– ΔCT^ with *RPL13A* as a housekeeping gene. Two-tailed unpaired *t*-test was used to analyzed differences between hNECs with and without IL-13 treatment. Data were presented as median with an interquartile range. Fold change was quantified with reference to untreated hNECs. Scale bar = 20 μm. hNECs, *n* = 6.

To better understand the effect of RV on TJs of inflammatory airway model, we next examine the nasal epithelial barrier integrity, remodeling (cilia and goblet cells) and immune responses of IL-13-treated hNECs against high dose of acute RV infection. Firstly, we found that RV progeny production and viral RNA expression were significantly increased with or without IL-13 treatment. Interestingly, RV progeny production and viral RNA expression were significantly lower in IL-13-treated hNECs as compared to RV-infected hNECs without IL-13 (all *P* = 0.031; Figures 5A,B). Meanwhile, AlamarBlue assay showed no cellular toxicity in RV infection and IL-13 treatment (Supplementary Figure E3).

Rhinovirus infection showed reduced *Cldn3* mRNA expression in both IL-13-treated and untreated hNECs, albeit not statistically significant (*P* = 0.219 and 0.438; Figure 5C). With IL-13 stimulation, RV infection further reduced *Cldn3* mRNA expression as compared to untreated group (*P* = 0.063; Figure 5C), and IF staining showed a clear loss and interruption in Cldn3 staining from apical region to sub-junctional lateral membrane (Figure 5M). RV infection upregulated mRNA levels of *ZO-1* and *occludin* but in the presence of IL-13, we saw a redistribution of both proteins from junction regions to basolateral area in IF staining (Supplementary Figures E4A,B,F,G). We also investigated the effect of RV infection on the cell type of hNECs. RV infection upregulated the mRNA expression of *MUC5AC* in untreated hNECs but not in IL-13-treated hNECs as compared to their respective mock-infected controls (all *P* = 0.031; Figure 5D). Interestingly, RV infection downregulated *Foxj1* mRNA levels in both untreated and IL-13-treated hNECs (all *P* = 0.031; Figure 5E). Representative images of IF staining showed that RV infection increased expression of MUC5AC and reduced βIV-tubulin expression in IL-13-treated hNECs as compared to uninfected IL-13-treated hNECs while RV infection induced slight changes of expression in untreated group (Figures 5N,O).

We also examine the innate immune responses of IL-13-treated hNECs against acute RV infection. The mRNA expression of RV receptor *ICAM-1* was reduced in both IL-13-treated and untreated hNECs as compared to the respective mock-infected controls (all *P* = 0.031; Figure 5F). With IL-13 stimulation, RV infection further reduced *ICAM-1* mRNA expression as compared to infected hNECs without IL-13 (*P* = 0.094; Figure 5F). Similarly, RV pathogen recognition receptor *TLR3* was reduced in both IL-13-treated and untreated hNECs as compared to the respective uninfected controls (all *P* = 0.031; Figure 5G). Additionally, RV infection increased the mRNA expression of antiviral type III IFN (*IFN-*λ*1*) and chemokine *CXCL10* in both IL-13-treated and untreated hNECs (all *P* = 0.031; Figures 5H,I). However, IL-13-treated hNECs has reduced capacity for upregulation of antiviral responses against RV infection as compared to RV infection in untreated hNECs as showed by lower extent of mRNA upregulation for both *IFN-*λ*1* and *CXCL10* (*P* = 0.063 and 0.031; Figures 5H,I). Moreover, RV infection upregulated mRNA expression of *IL-25* and *IL-33* only in untreated hNECs but not in IL-13-treated group (*P* = 0.031; Figures 5J,K), whereas *TSLP* mRNA was upregulated in both IL-13-treated and untreated groups (all *P* = 0.031; Figure 5L). With IL-13 stimulation, RV infection further increased *TSLP* mRNA expression as compared to untreated group (*P* = 0.063; Figure 5L). Similar to *IL-25* and *IL-33*, RV infection increased the mRNA expression of type-2 cytokine *IL-13* in untreated but not IL-13-treated hNECs (*P* = 0.031; Supplementary Figure E4D). There was no significant change in mRNA expression of *IL-5* and *IL-17A* (Supplementary Figures E4C,E) while *IL-4* mRNA expression was undetectable (data not shown).

**FIGURE 6:**
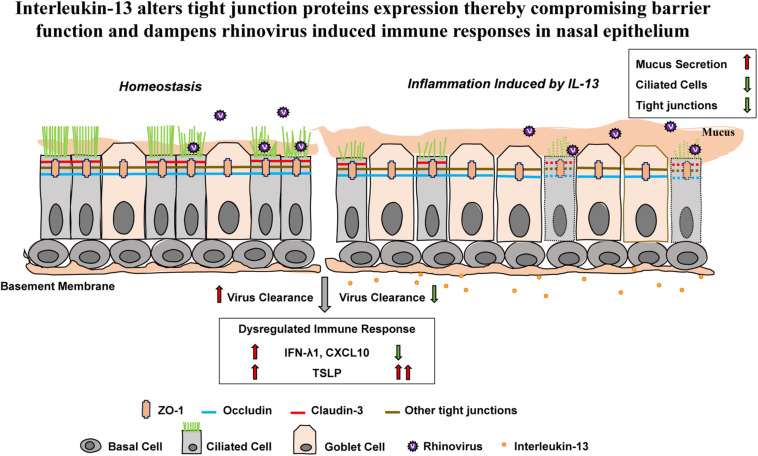
The effect of IL-13 on nasal epithelial barrier dysfunction and immune responses against RV infection in hNECs. Altered hNECs composition (cilia loss and mucus overproduction) in the presence of IL-13 is associated with the reduced expression of TJ proteins. Different TJ proteins are located in different types of nasal epithelial cells whereby ZO-1 and occludin are expressed in both ciliated cells and goblet cells while claudin-3 is only expressed in ciliated cells. RV infection which only targets ciliated cells induced minimal changes to TJs dysfunction. However, hNECs predisposed with IL-13 stimulation impaired the capacity for interferon activation and chemokine signaling. Moreover, the upregulation of *TSLP* expression in IL-13-treated hNECs suggests that RV infection in nasal epithelium predisposed with type-2 cytokine could lead to enhanced allergic inflammation which may further drive inflammation during RV-induced exacerbation of disease. The green arrow indicates downregulation and the red arrows indicate upregulation.

These results show that proinflammatory cytokine IL-13 disrupts epithelial barrier as well as reduces capacity for antiviral response against acute RV infection in hNECs ALI culture (Figure 6).

## Discussion

Our study demonstrates an impaired epithelial barrier function in CRSwNP patients along with decreased protein and mRNA expression of ZO-1, occludin and Cldn3. Long-term exposure of IL-13 altered hNECs composition in conjunction with nasal epithelial barrier dysfunction and enhanced mucosal inflammation, as well as reduced capacity for antiviral response against acute RV infection. These observations extend the knowledge of dysregulation of TJs in chronic airway diseases such as CRSwNP. Most importantly, ALI-cultured hNECs are shown to form functional barriers similar to that found in nasal biopsies. Using the IL-13-matured hNECs model, we have demonstrated that pro-inflammatory mediator IL-13 regulates the formation TJs and thereby disrupts the nasal epithelial barrier functions.

Nasal epithelial cells form a functional barrier which is mainly regulated by TJs. Recent studies have highlighted multiple defective TJs in patients with chronic airway diseases (Buckley and Turner, 2018) and reported that ZO-1, claudin-1, claudin-4, and occludin contributed to the leaky barrier of airway epithelium (Jacob and Gaver, 2012; Gan et al., 2013; Lee et al., 2016). In our study, we found that the expression of Cldn3, similar to ZO-1 and occludin, is significantly lower in sinonasal tissues of CRSwNP as compared to healthy controls. Moreover, we noticed that occludin and Cldn3 protein expression level negatively correlates with MUC5AC expression and positively correlates with βIV-tubulin expression in nasal biopsies specimens. It appears that alteration of nasal epithelial homeostasis during inflammation may impair TJs integrity. For this purpose, we used ALI-cultured hNECs with IL-13 stimulation which mimics the remodeling of nasal epithelium during chronic inflammation to explore the formation and disruption of TJs and thereby the IL-13-induced barrier damage.

We demonstrated that long-term exposure of hNECs to IL-13 results in overproduction of mucus and cilia loss along with impaired epithelial barrier function which is evident by increased epithelium permeability and decreased TEER. ZO-1, occludin and Cldn3 structures were also disrupted by IL-13 treatment during hNECs differentiation. In addition to enhanced inflammation, defective barrier function allows foreign substance to infiltrate the sinonasal submucosa and causes aggravation of airway inflammation and remodeling. Hence, TJs play an important in the pathophysiology of chronic airway disease. Taken together, these data indicate that impaired formation of TJs protein ZO-1, occludin and Cldn3 is largely associated with barrier dysfunction.

Although previous studies have examined TJs expression following various cytokine insults, differentiated epithelial cell lines or primary airway epithelial cells were commonly used (Lee et al., 2016). However, these cell models were not able to elucidate the processes of TJs formation during epithelium differentiation. Therefore, we investigate the effect of IL-13 on TJs protein formation during the time course of ALI cultivation. We found that positively stained ZO-1 and occludin were observed as early as Day 7 and 3, respectively at early stage of differentiation of hNECs with or without IL-13 treatment. Interestingly, *Cldn3* gene expression only increased when ciliated cells were first observed at Day 11 and is expressed only on ciliated cells of hNECs. Hence, the reduced levels of Cldn3 may be attributed to loss of cilia in airway epithelium of chronic inflammatory airway disease. Additionally, as key barrier function proteins, claudins serve as paracellular barrier (Gunzel and Yu, 2013) and are shown to have differential tissue-specific expression patterns which account for the differences in paracellular tightness and ion selectivity (Coyne et al., 2003; Bauer et al., 2014; Markov et al., 2015). Although Cldn3 is expressed in the airways, its role in airway barrier function has not been fully defined. Studies in lower airways have demonstrated that the expression level and function of Cldn3 are not comparable between type-I and type-II alveolar cells (Wang et al., 2003; Yu et al., 2005; Mitchell et al., 2011). Therefore, consistent with study which reported varying TJ composition in different airway epithelial cell type, the differential expression of Cldn3 in our study implies that Cldn3 may be involved in regulation of epithelial barrier as well as in epithelial differentiation. However, the involvement of Cldn3 in ciliogenesis during differentiation is currently unknown. Thus, further studies will be needed to investigate how TJ composition and epithelial cell differentiation interrelate for regulation of barrier permeability within stratified epithelia.

As the nasal airway is the primary target site for most respiratory viral infections, impaired epithelial barrier could lead to greater susceptibility against viral infection and dysregulation of host innate immune responses (Vareille et al., 2011). RV is the most prevalent respiratory virus in CRSwNP patients and is the most commonly associated with exacerbation of chronic airway disease (Sajjan et al., 2008; Kennedy et al., 2012; Cho et al., 2013). As RV infection may have significant implications in regulating the epithelial barrier function and mucosal inflammation of CRSwNP, we further investigate the effect of RV infection on TJs of IL-13-matured hNECs and the effects of IL-13 on the host responses of hNECs against RV infection. We found that the altered hNECs composition (cilia loss and mucus overproduction) in the presence of IL-13 is associated with the reduced RV replication (viral RNA level) and viral particle formation as compared to RV infection without IL-13 at the same initial infectious dose. As our previous study found that RV almost exclusively infected ciliated cells but not goblet and basal cells in *in vitro* hNECs (Tan et al., 2018), cilia loss due to IL-13 may impede RV infection by reducing target cells for viral replication. However, despite lower viral replication and production, RV infection of IL-13-treated hNECs worsened the mucociliary function by further inducing loss of cilia as shown by reduction of *Foxj1* mRNA level and IF staining. While studies have reported that RV infection disrupted TJs in primary airway epithelial cells (Sajjan et al., 2008; Yeo and Jang, 2010), our data showed that RV infection induced minimal alteration to TJs proteins in hNECs with and without IL-13 treatment. In addition, we investigated the effects of IL-13 on innate immune responses of hNECs against RV infection. We found that viral entry receptor *ICAM-1*, RV-induced host pathogen sensor (TLR3) and antiviral immune responses (IFN-λ1 and CXCL10) were upregulated in both untreated and IL-13-treated hNECs, suggesting that RV infection induced immune surveillance and antiviral responses even in inflammatory hNECs model. However, the capacity for interferon activation and chemokine signaling were impaired when hNECs is predisposed with IL-13 environment as shown by lower *IFN-λ1* and *CXCL10* expression as compared untreated hNECs. In contrast, RV-induced greater upregulation of *TSLP* expression in IL-13-treated hNECs suggesting that RV infection in nasal epithelium predisposed with type-2 cytokine environment could lead to enhanced allergic inflammation which may further drive inflammation during RV-induced exacerbation of disease. Our current study investigated the effects of mild respiratory virus RV-16, which is commonly associated to chronic respiratory disease exacerbations, on epithelium barrier function and immune capacity of hNECs in type-2 cytokine environment. While RV infection induced minimal change to TJs dysfunction, the impairment of efficient antiviral response in nasal epithelium may be attributed to IL-13-induced change in hNECs composition. More pathogenic respiratory viruses could be studied using this model to assess the epithelia barrier function and antiviral responses of chronic inflammatory airway disease.

In conclusion, IL-13, a typical type-2 cytokine, contributes to diminished barrier function and airway inflammation that are seen in CRSwNP patients. Knowledge about the dysregulation of TJs will help to better understand the pathophysiology of CRSwNP and define the specific mechanisms that link allergic inflammation and antiviral responses, which could lead to new strategies for the prevention and treatment of the disease.

## Materials and Methods

### Study Patients

Nasal biopsy specimens were recruited from the Department of Otolaryngology, First Affiliated Hospital of Nanchang University; the Department of Otolaryngology, National University Hospital of Singapore; and the Department of Otolaryngology, Second Hospital of Shandong University. 70 CRSwNP patients who underwent functional endoscopic sinus surgery were recruited. CRSwNP diagnoses were made according to the current European position paper on rhinosinusitis and nasal polyps (EPOS 2020) (Fokkens et al., 2020). 25 control subjects who did not have a history of sinonasal inflammation and inferior turbinate (IT) tissues were taken from patients who underwent septal plastic surgery. The ethics approval was obtained from the institutional review boards of the participating hospitals in China (2019124) and National Healthcare Group Domain Specific Review Board of Singapore (DSRB D/11/228 and IRB 13–509). All participants gave informed consent to participate in the study. The clinical characteristics of the study subjects were shown in Table 1.

**TABLE 1:** Characteristics of subjects providing sinus tissue.

	Healthy control subjects	Patients with CRSwNP	*P* value*
Sample size (No.)	25	70	NA.
Gender (M/F)	16/9	47/23	0.969
Age (y, Mean ± SEM)	35.84 ± 3.52	40.25 ± 12.65	0.337
Atopy (N%)	3 (12%)	16 (23%)	0.355
Asthma (N%)	0 (0%)	11 (16%)	0.081
Smoker (N%)	2 (8%)	12 (17%)	0.390

NA, not applicable; M, male; F, Female. *P value was evaluated using the Fisher’s exact test. *P* < 0.05 was considered statistically significant.

### Cell Culture and IL-13 Stimulation

The hNECs were differentiated from human nasal epithelial stem/progenitor cells (hNESPCs) isolated from IT of healthy subjects (*n* = 9). The hNESPCs were transferred to an ALI system to form a pseudostratified layer within 4 weeks. Methods for culturing hNECs were described in previous paper (Li et al., 2014). IL-13 (10 ng/ml, R&D System, Minneapolis, MN, United States) was added in the medium on the first of ALI and medium with IL-13 were replenished every 2 days for 21 days until they were fully differentiated.

### Infection and Quantification of RV

Fully differentiated hNECs with and without IL-13 treatment were inoculated with RV16 (ATCC VR-283^®^) at a high dosage of MOI 10 and incubated at 33°C for 1 h. Apical wash and RV-infected and uninfected hNECs were harvested at 24 h post-infection. Plaque assay was performed as previously described (Tan et al., 2018). The plaque-forming unit (PFU) was calculated as follows: Number of plaques × dilution factor = number of PFU per 100 μl.

### TEER

The TEER of IL-13-matured hNECs was measured from Day 3 to 21 of ALI culture by using an EVOM voltammeter device with STX2 electrode (WPI, Sarasota, FL, United States). TEER was calculated by subtracting blank value. The total TEER (ohms⋅cm^2^) is presented by TEER measurement (ohms) × Area of a membrane (cm^2^).

### Barrier Function Assays

The TJ permeability assay was performed according to previous study by using surface biotinylation (Chen et al., 1997). Briefly, ALI-cultured hNECs were incubated with 1 mg/ml EZ-Link Sulfo-NHS-LC-biotin (557 Da; Thermo Fisher Scientific, Inc., Waltham, MA, United States) in DPBS for 45 min. After washing, the samples were fixed overnight with 4% PFA at 4°C, embedded in paraffin, and sectioned at a thickness of 4 μm. After antigen retrieval, sections were washed and blocked with goat serum, and then incubated for 30 min with streptavidin (Alexa Fluor^TM^ 488 conjugate, Thermo Fisher Scientific).

### Immunohistochemistry (IHC) and Immunofluorescence (IF) Staining

Immunohistochemistry and IF staining was performed for paraffin sections of nasal tissue, transwell membranes of ALI culture and cytospin clinical samples. The primary antibody information was showed in Supplementary Table E1. More details of antibodies and staining procedures are described in the Supporting Information.

### Total Fluorescence Intensity (TFI) Evaluation

Images of ZO-1, occludin, claudin-3 (Cldn3), MUC5AC, and βIV-tubulin on paraffin sections were captured at 400× magnification with a fluorescence microscope (Olympus IX51, Tokyo, Japan). Protein expression of these markers was analyzed using ImageJ software by calculating the value of positively stained area and the mean fluorescence intensity for each marker. Total fluorescence intensity (TFI) measurements were performed by multiplying the positive area by mean fluorescence intensity and corrected by subtracting the background autofluorescence.

### Immunoblotting

The preparation of the cell lysates, SDS-PAGE and western blot analysis were performed according to standard protocols. Equal loading for each sample was used to detect ZO-1, occludin, Cldn3, and glyceraldehyde 3-phosphate dehydrogenase (GAPDH). The final assessment was evaluated as ratio of target protein to housekeeping protein.

### RNA Isolation and Quantitative Real-Time PCR

Total RNA was extracted from frozen nasal tissues and hNECs using the mirVana miRNA Isolation Kit (Life Technologies, United States). Complementary DNA was synthesized in a 20 μl reaction volume from 1 μg total RNA using qScript^TM^ cDNA SuperMix (Quanta BioDesign) according to the protocol of manufacturer. Relative genes expression was detected using SYBR green gene expression assays and was normalized to 2^–ΔCT^ with *GAPDH* and *ribosomal protein L13a* (*RPL13A*) as a housekeeping gene. The gene primer sequences used were shown in Supplementary Table E2.

### Statistics

All data were analyzed with GraphPad Prism 7 software (GraphPad Software, La Jolla, CA, United States). Fisher’s exact test, Mann–Whitney *U* test and Wilcoxon signed-rank test were used to analyze differences between two groups. Two-way ANOVA was used to analyzed differences between hNECs with or without IL-13 treatment. Fold change was quantified with reference to untreated hNECs. Data were presented as Mean ± SEM or Median with interquartile range (25–75%). Correlation analysis was performed using Spearman r characteristic. *P* < 0.05 was considered statistically significant.

## Data Availability Statement

The raw data supporting the conclusions of this article will be made available by the authors, without undue reservation.

## Ethics Statement

The studies involving human participants were reviewed and approved by The Institutional Review Board and Research Ethics Committee of The First Affiliated Hospital of Nanchang University and The National Healthcare Group Domain Specific Review Board of Singapore. The patients/participants provided their written informed consent to participate in this study.

## Author Contributions

D-YW and JY designed the study. Z-QH and JL wrote the manuscript and performed most of the experiments. HHO performed the experiments and help to edit the manuscript. VTC provided RV infection and technical assistance. KST performed the experiments. TY, X-MZ, JW, Q-TY, and LS collected the clinical and hNECs samples. All authors have read and agreed to the final version of the manuscript.

## Conflict of Interest

The authors declare that the research was conducted in the absence of any commercial or financial relationships that could be construed as a potential conflict of interest.
