# Supplementary material for: Interleukin-13 Alters Tight Junction Proteins Expression Thereby Compromising Barrier Function and Dampens Rhinovirus Induced Immune Responses in Nasal Epithelium
Source: Front Cell Dev Biol. 2020 Sep 25;8:572749. doi: 10.3389/fcell.2020.572749 (PMC7546404; doi:10.3389/fcell.2020.572749)
Supplement: Supplementary file 1 [file Data_Sheet_1.PDF]

Supporting Information

Interleukin-13 alters tight junction proteins expression thereby compromising barrier function and dampens rhinovirus induced immune responses in nasal epithelium

Zhi-Qun Huang, MD[1,2,3†], Jing Liu, MD, PhD[2†], Hsiao Hui Ong, BSc[2], Tian Yuan, MD, PhD[2,4], Xiang-Min Zhou, MD[2,5], Jun Wang, MD[1], Kai Sen Tan, MD, PhD[2], Vincent T Chow, MD, PhD[6], Qin-Tai Yang, MD, PhD[4], Li Shi, MD, PhD[5], Jing Ye, MD, PhD[1*], De-Yun Wang, MD, PhD[2*]

[1]Department of Otolaryngology-Head and Neck Surgery, First Affiliated Hospital of Nanchang University, Jiangxi, China.

[2]Department of Otolaryngology, Yong Loo Lin School of Medicine, National University of Singapore, National University Health System, Singapore, Singapore.

[3]Department of Otolaryngology-Head and Neck Surgery, Affiliated Hospital of Jiujiang University, Jiangxi, China.

[4]Department of Otorhinolaryngology-Head and Neck Surgery, Third Affiliated Hospital, Sun Yat-sen University, Guangzhou, China.

[5]Department of Otolaryngology, Second Hospital of Shandong University, Jinan, China.

[6]Department of Microbiology and Immunology, Yong Loo Lin School of Medicine, National University of Singapore, Singapore.

†These authors have contributed equally to this work.

*** Corresponding author:**

De-Yun Wang, MD, PhD, Department of Otolaryngology, Yong Loo Lin School of Medicine, National University of Singapore, 1E Kent Ridge Rd, Singapore 119228. Tel: +65-67725373. E-mail: entwdy@nus.edu.sg

Jing Ye, MD, PhD, Department of Otorhinolaryngology, Head and Neck Surgery, First Affiliated Hospital of Nanchang University, No.17 Yongwaizheng Street, Nanchang, 330006, China. Tel: +86-13979161109. E-mail: yjholly@email.ncu.edu.cn

Supplemental Methods

IHC and IF staining

IHC and IF staining was performed for paraffin sections of nasal tissue, transwell membranes of ALI culture and cytospin clinical samples.

Antibodies was showed in Table E1.

IHC staining First, nasal biopsy tissues were fixed in formalin, embedded in paraffin and sectioned at 4μm with Leica microtome (Leica, Wetzlar, Germany). Then, the paraffin sections were deparaffinized, hydrated through graded alcohols. The sections were heated in a microwave oven for 10 minutes at 100°C with pH6 retrieval buffer (Dako A/S) for antigen retrieval. Endogenous peroxidase activity was blocked with 3% H_2O_2 for 5 minutes at room temperature. Non-specific immune reactivity was blocked with 10% goat serum for 30 minutes at room temperature. Slides were stained with anti-IL-13 primary antibody and incubated at 4°C overnight. The slides were then incubated with DAKO EnVision+System-HRP (Dako A/S) for 30 minutes at room temperature. Diaminobenzidine was used as substrate for color development. All slides were counterstained with hematoxylin (Sigma Aldrich), dehydrated with serial concentration of ethanol, hydrated with xylene and mounted with mounting medium (Dako A/S).

IF staining Double staining was performed in IF staining by using two different host species antibodies. The combinations of the primary antibodies include: ZO-1 with MUC5AC, ZO-1 with βIV-tubulin; occludin with MUC5AC, occludin with βIV-tubulin; Cldn3 with MUC5AC, Cldn3 with βIV-tubulin and βIV-tubulin with MUC5AC.

(1) Paraffin tissue sections were deparaffinization, rehydration and processed with pH6 retrieval buffer (Dako A/S) before blocking with 10% goat serum.
(2) hNECs from ALI cultures with or without cytokine/test compounds treatments were spun onto microscopic slides at 500 rpm for 5 minutes using a cytospin. Cell smears were fixed in cold acetone for 10 minutes at -20°C and were permeabilized with 0.1% TritonX-100 for 10 minutes at room temperature before blocking with 10% goat serum.
(3) Transwell membranes from ALI cultures with or without cytokine/test compounds treatments were fixed by formalin for 15 minutes. After washing by PBS, the cells were then permeabilized with 0.1% TritonX-100 for 10 minutes at room temperature before blocking with 10% goat serum.

Paraffin tissue sections, cytospin samples and transwell membranes were incubated with primary antibodies at 4°C overnight and then incubated with Alexa Fluor 488 or 594 conjugated secondary antibodies (goat-anti mouse or rabbit IgG (H+L), molecular probes, Carlsbad, CA) at 1:500 in the dark for 1 hour at room temperature and followed by mounting the slides with Antifade reagent with DAPI (molecular probes, Carlsbad, CA).

Cell Viability Assay

Cell viability was assessed using the AlamarBlue cell viability assay (Thermo Fischer, Scoresby, VIC, Australia). hNECs were dissociated by trypsinization and 10% AlamarBlue was added to sample. 100ul of each sample was added into each well in triplicates and plates were incubated at 37°C for 2 hours. Absorbance was measured at 570nm and 600nm on a Tecan Infinite M200 Plate Reader and percentage reduction was calculated according to the following equation:

$$\text{Percent reduced} = \frac{(\varepsilon_{OX})\lambda_2 A\lambda_1 - (\varepsilon_{OX})\lambda_1 A\lambda_2}{(\varepsilon_{RED})\lambda_1 A'\lambda_2 - (\varepsilon_{RED})\lambda_2 A'\lambda_1} \times 100$$

Supplemental Table Legend

Table E1. Antibodies for staining.

Antibody	Type	Dilution		Catalog number	Company
		IF/IHC	WB		
ZO-1	pAb	1/400	1/1000	HPA001636	Sigma
ZO-1	mAb	1/400		33-9100	Thermo Fisher Scientific
Occludin	pAb	1/400	1/1500	71-1500	Thermo Fisher Scientific
Cldn3	pAb	1/400	1/1000	ab15102	Abcam
MUC5AC	pAb	1/500	1/500	sc-20118	Santa Cruz Biotechnology
MUC5AC	mAb	1/500		ab24071	Abcam
βIV-tubulin	mAb	1/500		ab11315	Abcam
Foxj1	pAb		1/500	HPA005714	Sigma
IL-13	pAb	1/1500		ab106732	Abcam
GAPDH	mAB		1/10000	ab8245	Abcam

IF: Immunofluorescence; IHC: Immunohistochemistry; WB: Western blotting; pAb: rabbit polyclonal antibody; mAb: mouse monoclonal antibody.

Table E2. Primer sequences used for RT-qPCR.

Gene	Forward primer	Reverse primer
ZO-1	TTGTCTTCAAAAACTCCCAC	GACTCACAGGAATAGCTTTAG
Occludin	GGACTGGATCAGGGAATATC	ATTCTTTATCCAAACGGGAG
Cldn3	CTGCATGGACTGTGAAAC	AAAATATCAAGTGCCCCTTC
MUC5AC	AATGGTGGAGATTTTGACAC	TTCTTGTTCAGGCAAATCAG
Foxj1	GTGAAGCCTCCCTACTC	TTCTTGTTCAGGCAAATCAG
RV-16	GCACTTCTGTTTCCCC	CGGACACCCAAAGTAG
ICAM-1	ACCATCTACAGCTTTCCG	TCACACTTCACTGTCACC
TLR3	AGATTCAAGGTACATCATGC	CAATTTATGACGAAAGGCAC
IFN-λ1	CAGGTTCAAATCTCTGTCAC	AACTCCAGTTTTTCAGCTTG
CXCL10	AAAGCAGTTAGCAAGGAAAG	TCATTGGTCACCTTTTAGTG
IL-25	CAGGTGGTTGCATTCTTGGC	GAGCCGGTTCAAGTCTCTGT
IL-33	GCTGGGAAATAAGGTGTTAC	CCAGAAGTCTTTTGTAGGAC
TSLP	AAAGTACCGAGTTCAACAAC	GTAGCATTTATCTGAGTTTCCG
IL-4	TCACATTGTCACTGCAAATC	CCTTCTCAGTTGTGTTCTTC
IL-5	AACTGTGCACTGAAGAAATC	CTAGGAATTGGTTTACTCTCC
IL-13	ATCACCCAGAACCAGAAG	ATGCAAGCTGGAAAACTG
IL-17A	GTATGAGAAAAGTTCAGCCC	TGGTTACGATGTGAAACTTG
GAPDH	ACAGTTGCCATGTAGACC	TTGAGCACAGGGTACTTTA
RPL13A	GTCTGAAGCCTACAAGAAAG	TGTCAATTTTCTTCTCCACG

RT-qPCR: Quantitative Real-Time Polymerase Chain Reaction; *ICAM-1: intercellular adhesion molecule 1; TLR3: toll-liker receptors 3; IFN-λ1: interferon lambda 1; CXCL10: C-X-C motif chemokine ligand 10; TSLP: thymic stromal lymphopoietin.*

Supplemental Figure Legend

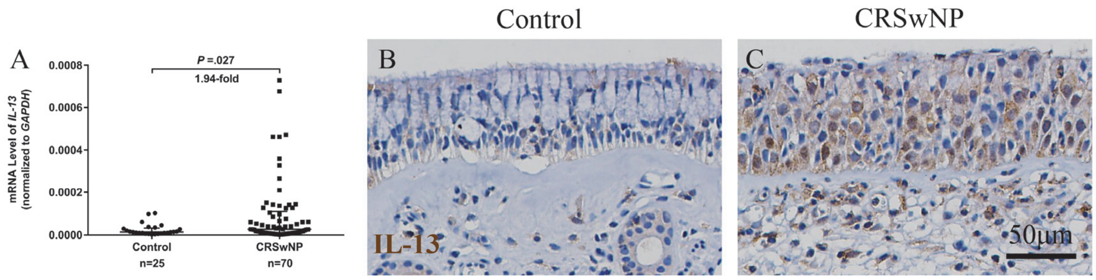

Figure E1. The expression level of IL-13 in CRSwNP patients. *IL-13* mRNA level was significantly increased in CRSwNP patients (**A**). Representative immunohistochemistry images showed that IL-13 was oversecretion in CRSwNP patients (**B-C**). Relative expression of the target gene was normalized to $2^{-\Delta CT}$ with *GAPDH*. Statistical analysis was calculated using the Mann-Whitney U test. Data were presented as median with an interquartile range. Scale bar=50 μm.

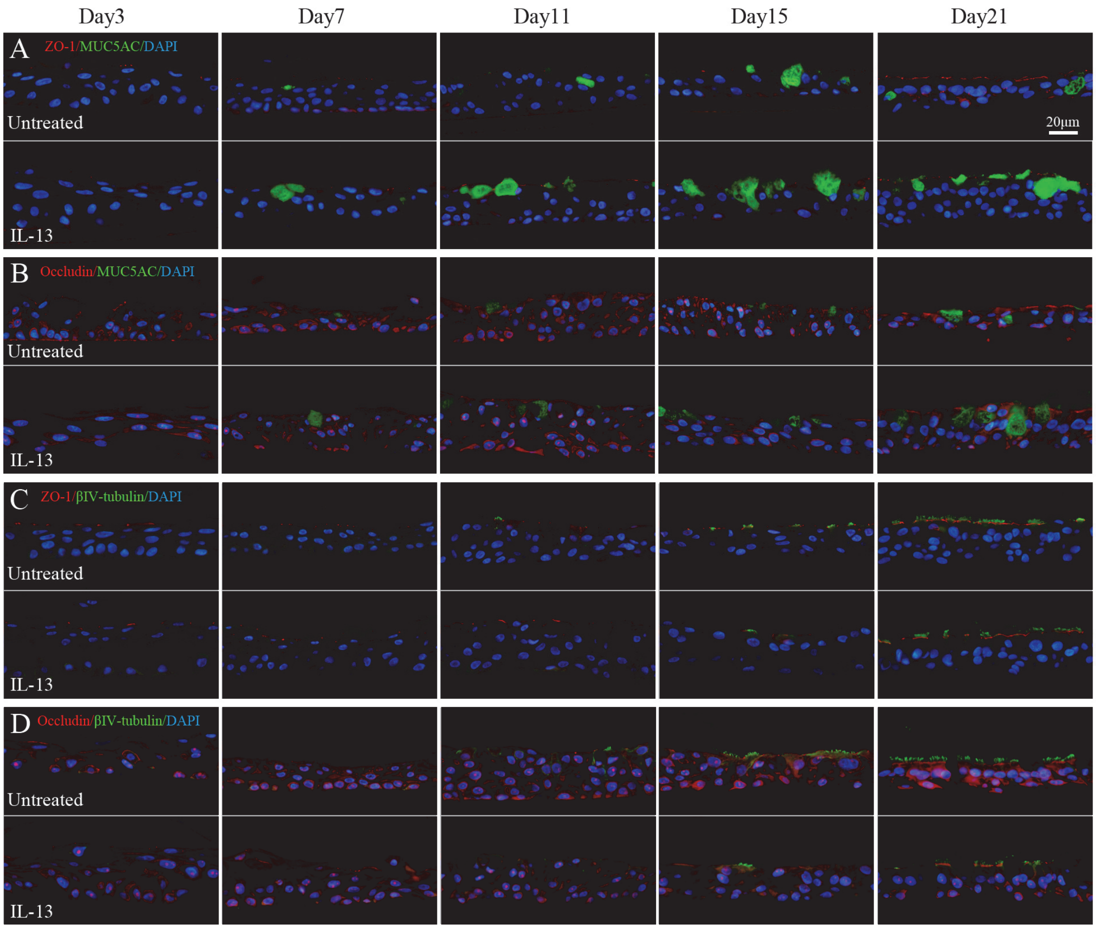

Figure E2. Expression of TJ proteins during hNECs differentiation. Positive staining of ZO-1 and occludin was observed at cell to cell contact sites even in early stage of differentiation. Localization pattern was not linear but fragmented at cell to cell boundaries after IL-13 treatment (**A-D**). Scale bar=20 μm.

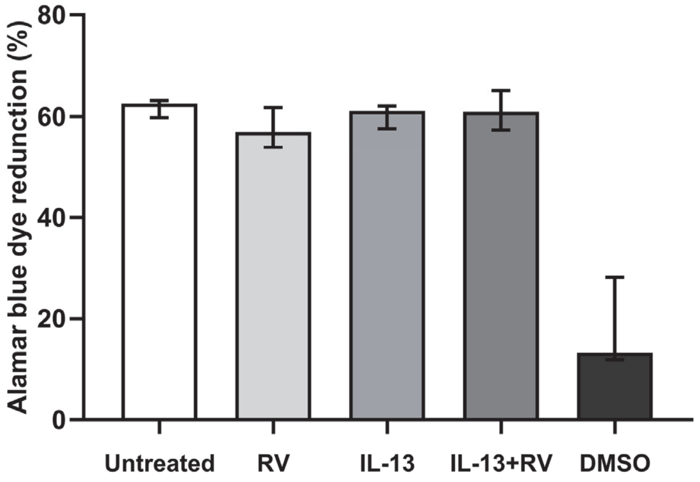

Figure E3. The cellular toxicity level of hNECs after RV and IL-13 treatment. AlamarBlue assay showed no cellular toxicity in RV infection and IL-13 treatment. Two-tailed unpaired t-test was used to analyzed differences between hNECs with and without IL-13 treatment. Data were presented as median with an interquartile range. hNECs, n=3.

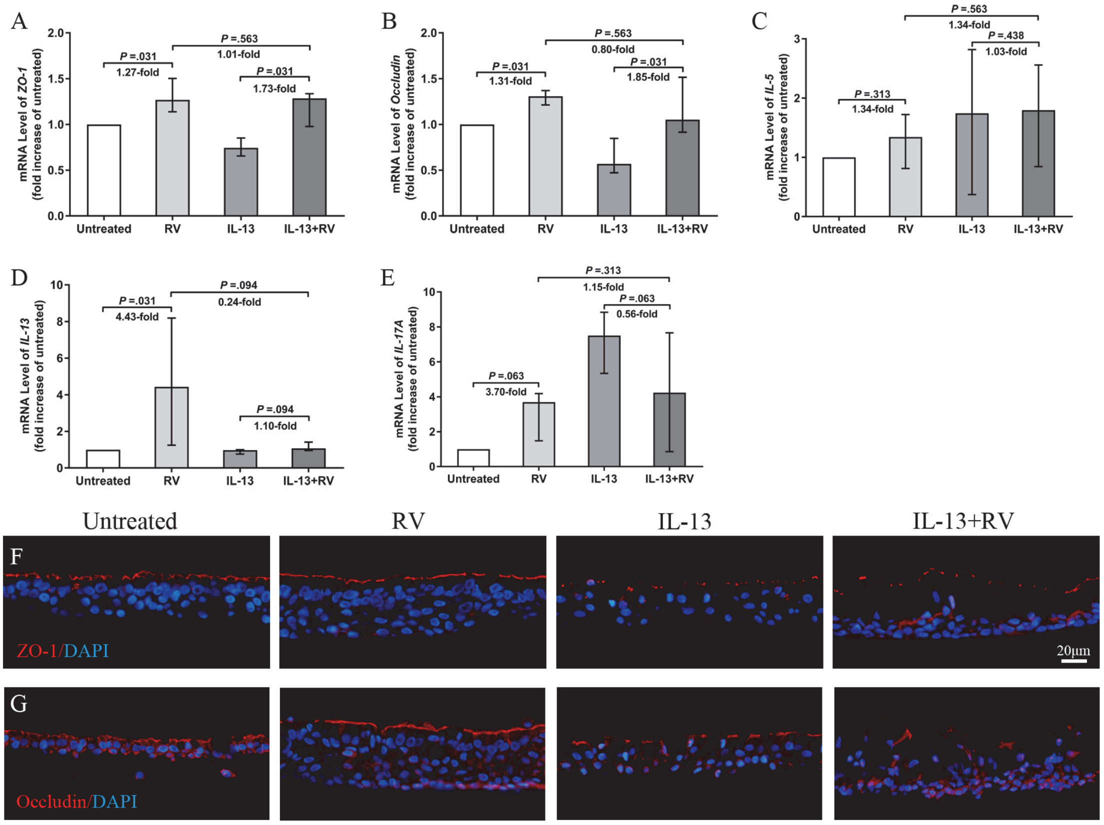

Figure E4. Effects of RV infection on TJs and innate immune response on IL-13-treated hNECs. *ZO-1* and *occludin* mRNA levels were increased in both untreated and IL-13-treated hNECs. RV significantly regulated *ZO-1* and *occludin* mRNA levels in both untreated and IL-13-treated hNECs (**A-B**). There was no significant change in mRNA expression of *IL-5* (**C**) while *IL-4* mRNA expression was undetectable (data not shown). RV infection significantly increased the mRNA expression of *IL-13* in untreated but not IL-13-treated hNECs. RV significantly upregulated mRNA expression of *IL-13* for untreated hNECs but not IL-13-treated hNECs (**D**). There was no significant change in mRNA expression of *IL-17A* (**E**). Representative images of IF staining showed that RV infection in both untreated and IL-13-treated hNECs induced slight increase in expression of ZO-1 and occludin (**F-G**). The relative target gene was normalized to $2^{-\Delta CT}$ with *RPL13A* as a housekeeping gene. Two-tailed unpaired t-test was used to analyzed differences between hNECs with and without IL-13 treatment. Data were presented as median with an interquartile range. Fold change was quantified with reference to untreated hNECs. Scale bar=20 μm. hNECs, n=6.
